# Default Mode Network, Meditation, and Age-Associated Brain Changes: What Can We Learn from the Impact of Mental Training on Well-Being as a Psychotherapeutic Approach?

**DOI:** 10.1155/2019/7067592

**Published:** 2019-04-02

**Authors:** Ricardo Ramírez-Barrantes, Marcelo Arancibia, Jana Stojanova, Mauricio Aspé-Sánchez, Claudio Córdova, Rodrigo A. Henríquez-Ch

**Affiliations:** ^1^Escuela de Tecnología Médica, Universidad Andrés Bello, Quillota 980, 2531015 Viña del Mar, Chile; ^2^Interdisciplinary Centre for Health Studies (CIESAL), Universidad de Valparaíso, Angamos 655, 2540064 Viña del Mar, Chile; ^3^Biomedical Research Centre (CIB), Universidad de Valparaíso, Angamos 655, 2540064 Viña del Mar, Chile; ^4^School of Medicine, Universidad de Valparaíso, Angamos 655, 2540064 Viña del Mar, Chile; ^5^División de Neurociencias (NeuroCICS), Centro de Investigación en Complejidad Social (CICS), Facultad de Gobierno, Universidad del Desarrollo, Santiago, Chile; ^6^Laboratorio de Estructura y Función Celular, Escuela de Medicina, Facultad de Medicina, Universidad de Valparaíso, Hontaneda 2664, 2341386 Valparaíso, Chile; ^7^Laboratorio de Neurociencia Cognitiva y Social, Facultad de Psicología, Universidad Diego Portales, Chile; ^8^Laboratory of Cognitive Neuroscience, Interdisciplinary Center for Neuroscience, School of Medicine, Pontificia Universidad Católica de Chile, Santiago, Chile

## Abstract

Aging is a physiological process accompanied by cognitive decline, principally in memory and executive functions. Alterations in the connectivity of the default mode network (DMN) have been found to participate in cognitive decline, as well as in several neurocognitive disorders. The DMN has antisynchronic activity with attentional networks (task-positive networks (TPN)), which are critical to executive function and memory. Findings pointing to the regulation of the DMN via activation of TPN suggest that it can be used as a strategy for neuroprotection. Meditation is a noninvasive and nonpharmacological technique proven to increase meta-awareness, a cognitive ability which involves the control of both networks. In this review, we discuss the possibility of facilitating healthy aging through the regulation of networks through meditation. We propose that by practicing specific types of meditation, cognitive decline could be slowed, promoting a healthy lifestyle, which may enhance the quality of life for the elderly.

## 1. Aging and Mental Diseases

Life expectancy has increased in most of the world's population, mainly in developed countries. According to data from the United Nations, the proportion of persons over 60 years will be 20% of the total population in 2050, doubling the current elderly population [1]. While the mechanisms underlying the biological process of aging are not completely understood, in general terms aging is characterized by a progressive decrease of physiological function and an increased vulnerability to death [2].

Regarding cognitive and neural changes, aging involves a progressive decline in specific brain networks and cognitive processes, such as memory encoding and retrieval, and executive control functions, all of which are progressively impaired [3]. Executive functions are responsible for flexible and adaptive behaviours [4]; in other words, they are “the means by which our brain optimizes the flexible use of limited cognitive resources to currently prioritized tasks” [5]. Memory, on the other hand, constitutes the ability of living organisms to retain and utilize acquired information. Both memory and executive functions include subprocesses that are differently affected by aging. For instance, long-term memory is usually impaired in elder people, but the retrieval of word meaning is not [6]. Memory and executive functions are interrelated cognitive processes, and both play a critical role in complex situations of everyday life [4]. Executive functions usually control acts of remembering: for example, guiding event retrieval by recalling the sources of the information that subjects aim to remember [6, 7]. Along this line, memory decline in aging could stem from a disruption of the executive functions that influence memory, and not necessarily to a disruption in memory itself [6, 8]: if a subject tries to fruitlessly recall a friend's name, it could imply damage both in memory storage and in executive functions, by being unable to focus on the process of retrieval.

The relationship between cognitive function and neuronal connectivity has been studied in several recent investigations. Particularly in aging, an increase of incoherent function in specific brain networks diminishes the efficiency and efficacy of information processing in the brain [3]. One of the networks that have been reported to be altered in old age include the default mode network (DMN) [9, 10], which is associated to mind wandering or spontaneous thought, self-reflective thinking, inner speech, incidental self-processing, stimulus-independent thought, momentary attentional lapses, task-unrelated thought, and autobiographical memory, among other processes. Other networks associated with high cognitive functions, like the attentional and task-positive networks (TPN), which include the dorsal and ventral frontoparietal attentional networks [11–13], are also modified through aging [3, 14–17]. On the other hand, lifestyle choices across the lifespan contribute positively to the process of aging [18–20]. Chronic stress, anxiety, or depression, for example, are common mental health conditions [21, 22] that accelerate many of the detrimental aspects of aging, such as cognitive decline, memory loss, and disruption of the DMN and attentional networks [23, 24].

Some reports indicate that abnormal functioning of the DMN could predispose individuals to mental diseases, like depression, anxiety, attention deficit, and posttraumatic stress disorders [23]. All of these factors, in the long term, affect the quality of life in the elderly. Prolonged periods of untreated mental illness over the lifespan, together with the neurological decline associated with aging, may have a detrimental effect on the quality of life of the elderly person. Our lifestyle not only affects our present mental state and brain function, but also influences how aging unfolds [18–20]. In this context, developing strategies that decrease the damaging aspects of aging, as well as of neurodegenerative and neuropsychiatric disorders, is a priority for promoting healthy aging. Enhancing cerebral resilience through pharmacological and nonpharmacological treatments may represent one approach. In this sense, we propose the regulation of the DMN and TPN as a means of promoting neuroprotection and optimizing aging.

## 2. Default Mode Network, Aging, and Mental Disease

The DMN is a member of a set of coherent fluctuations of brain activity called resting-state networks (RSN), which are activated when an individual is not engaged in any activity or superior cognitive process [25–27]. The RSN were initially described in functional magnetic resonance imaging studies, in which the interest was not the response to stimuli, but the brain activity that occurred when the subject was at rest [27, 28]. The importance of these networks has been discovered during the last two decades, with several interesting findings, such as brain energy consumption at rest. It has been reported that, in an average adult human, the intrinsic activity of the brain at rest represents 20% of all energy consumed, much more than other fundamental organs, such as the heart or liver [29]. Interestingly, when the type of brain activity changes, for example, while performing a task, energy consumption is slightly greater than the resting state (5% or less [29, 30]). Finally, from the total energy utilized by the brain in the resting state during wakefulness, 80% is used in neuronal firing and glutamate and GABA recycling [31]. These data suggest that the majority of brain energy consumption is related to the maintenance of basal activity and neuronal communication, which probably include the RSN, among others. In the same vein, numerous studies propose a fundamental role of the RSN, and especially the DMN, with normal brain function across the lifespan. A relationship between the RSN and various neurological and psychiatric conditions has been established [32–36], as have changes in RSN patterns during normal aging [37] and in mild cognitive impairment [38]. Finally, genetic studies have shown that RSN patterns have a hereditary component [38], and animal studies suggest that RSN are phylogenetically conserved in primates [38] and rats [39].

Among the different RSN, the DMN has gained special prominence due to its attenuation during active tasks [40]. The DMN is characterized by the synchronous activation of several separated regions in the brain, including the medial prefrontal cortex, posterior cingulate cortex, precuneus, inferior parietal lobule, and inferolateral temporal cortex [23, 27, 41].

The DMN shows a high level of simultaneous activation during rest, while their activity diminishes during the performance of goal-directed tasks [27, 42, 43]. These results come from a classical approach that compares activity patterns within, and interconnectivity between, DMN brain regions during rest versus when commencing a goal-directed activity that modulates this network. However, while most studies indicate that activity is similar between rest and task, in general DMN activity seems to be attenuated rather than extinguished when we are on task versus off task. This attenuation becomes an activation transition between DMN and TPN when subjects change from a rest condition (passive fixation with eyes open) to common *N*-back memory-updating tasks. These results suggest that the DMN is suppressed during task execution. Along the same line, DMN activation may be detrimental to task performance, evidencing an anticorrelation between regional changes in activity within the DMN and task performance [44–46]. More specifically, there are changes in activation and connectivity between DMN and frontoparietal (FP) networks when subjects perform a basic memory-updating task (0-back task), or a single detection task, versus when they perform a more difficult task (2-back task). These results indicate that as the task complexity increases from 0-back to 2-back, FP activation and within-network connectivity also increased, whereas DMN activation and within-network connectivity decreased [46, 47]. Therefore, connectivity decreases within the DMN is related to the cognitive demands from the task [25, 48, 49], which would be evidence about neural transitions between states related to the change in activation linked to the load or complexity of the task.

Decomposition of connectivity data, in both humans and macaque models, shows that one extreme of the principal gradient of the brain's connectivity is constituted by regions associated with sensory and motor functions, while the other extreme is constituted by the cortical regions that define the DMN [50]. Hence, DMN cortical structures show greater geodesic distance from the sensory and motor cortices. This enables the DMN to process information that is not domain specific to immediate sensory inputs, constituting a hub of multimodal representational information [50].

The DMN is also known to support self-referential processing, being the neurophysiological correlate of autobiographical memory, self-reflective thinking [27, 40, 51], envisioning of future events, considering the thoughts and perspectives of others [27, 52], and mind wandering [53]. All of these cognitive processes, according to studies that relate the activity of DMN to metacognitive processes, could be important in building and updating internal models of the world, based on memories about oneself or others. Despite the significance of the activation of this network, the function of the DMN is not fully understood. Previous studies indicate that the inability to suppress the DMN can lead to attentional lapses [54], such as episodes related to decreased performance in attentional tasks. These episodes have been associated with behavioral variability across groups, and this variability, in turn, has been proposed to be a marker of mind wandering [55]. Furthermore, depression, anxiety, stress, and attention deficit disorder are conditions characterized by a diminishing state of vigilance and an increase of inner dialogue toward negative ruminant thoughts [23]. Something distinctive occurs in neuropsychiatric and neurological diseases, such as schizophrenia and epilepsy, which are not always associated with an inability to suppress DMN activity [41, 56–60]. Neuropsychiatric disorders, such as schizophrenia, appear to be related to antagonistic activity during tasks that demand attention [61]. In Alzheimer's disease, on the other hand, the phenomenon is accompanied by a decreased activity and connectivity of the DMN, probably due to a decreased metabolism and physiological disruptions from plaque deposition [32, 62]. In task-related performance, older individuals exhibit a reduced deactivation of the DMN or an increase in its baseline activity [62–65], indicating that an inability to inhibit or shift from the default mode to task-activated attentional networks results in lower cognitive performance [63, 65]. Therefore, the balance of the activation and deactivation of the DMN appears to be important in maintaining healthy brain function, including executive functions, memory, and attention. All these data have been used to propose the DMN as a candidate biomarker of mental diseases [41]. We suggest that DMN activity could constitute a model for understanding the dynamics of cognitive decline in elderly people. In addition, we suggest that its regulation could be achieved by specific meditation practices and used as a way to promote optimal aging and prevent neurological and neuropsychiatric diseases.

## 3. Meta-Awareness: Attention Network vs. DMN

The DMN is a network with coherent activation during periods of rest, i.e., when brain activity related to attention is spontaneously oriented to internal thoughts, without awareness of theme [66, 67]. Consequently, in healthy individuals DMN activity correlates negatively with the goal-oriented TPN [44, 68]. The latter, opposite networks, are preferentially active in attention-demanding tasks [69], when the brain is focused on a cognitive task (internal or external).

The attentional network includes the lateral prefrontal cortex, premotor cortex, lateral parietal regions, occipital regions, anterior cingulate cortex, and insula [67]. From the point of view of cognitive neuroscience, the attentional system could be divided into three subsystems that perform different but interrelated functions: (i) alerting, (ii) orientation, and (iii) conflict monitoring [70]. These divisions were established by Posner and Petersen in the early ‘90s, based on behavioral studies of normal adults or patients with different forms of brain injury. The original review suggested that this classification is based on three basic concepts of the attention system: first, the brain's attention system is anatomically separated from other processing systems, such as the motor or sensory systems; second, attention involves diverse anatomical areas of the brain; and third, the areas involved in attention perform different functions and can be specified in cognitive terms [70, 71]. This classification has evolved to include other elements, such as self-regulation [71], but the three basic elements are considered fundamental for the understanding of how attention works [12, 71]. Following this classification, the following functions were determined: (i) alerting or vigilance of an impending stimulus, which involves the thalamus and the right frontal and right parietal cortex, maintaining a state of high sensitivity to incoming stimuli [72, 73]; (ii) orientation or selection of relevant information from multiple stimuli, which involves the superior parietal cortex, temporal parietal junction, frontal eye fields, and superior colliculus [71–73]; and (iii) monitoring and resolution of conflict between computations in different neural areas (executive attention) [74], which includes the anterior cingulate cortex, lateral ventral cortex, prefrontal cortex, and basal ganglia. This network is also especially important in the detection of cognitive, homeostatic, or emotional subjective events, and it provides signals to the executive network to act in accordance with the current objective [71, 72]. However, when the attentional stream is abruptly interrupted and lacks goal-directed stimuli, for instance, during mind-wandering episodes, the DMN shows a high degree of functional connectivity between regions. This default activity has been related to states in which a subject is awake and alert, but not actively involved in an attention-demanding or goal-directed task [27, 43]. This suggests the existence of an interplay between the DMN and attentional networks' activities. Success in goal-oriented activities seems not to necessarily require the absence of mind wandering, but the individual's ability to detect when the mind is not on task, in order to reorient attention back [67]. Therefore, the activation of the circuitry associated with attention is critical not only in focusing on an object of attention, but also in noticing when the attention is focused or not on a particular task. In this way, an individual can detect when the mind is disengaged from a task and starts to wander. This process is called meta-awareness (“metaconsciousness” or “metacognitive awareness”) and is defined as one's explicit knowledge of the current contents of thought, feelings, and perceptions [75, 76]. Thus, as meta-awareness is the mental ability that strengthens the capacity to be aware of the internal and external world, it could be used to intentionally initiate, direct, and/or sustain attentional processes [75].

Interestingly, the anticorrelation of the DMN and TPN, and the nature of TPN, suggests a simple strategy to control the DMN, potentiated by meta-awareness. This could be a useful intervention as it implies a modification of brain activity through an individual's regulation of their behavior.

## 4. Meditation: Voluntary Control of Brain Networks

One of the simplest techniques that alter patterns of brain activity of the DMN and TPN is meditation [41]. Meditation can be defined as a form of mental training that aims to improve psychological capacities, such as attentional and emotional self-regulation, perspective taking, and meta-awareness [74, 75, 77]. This includes several methods, organized under the concept of contemplative practices [75]. As Dorjee suggests, contemplative practices could be defined as several techniques that promote a metacognitive self-regulatory capacity of the mind, modulated by motivational, intentional, and contextual factors of contemplative practices [78]. This ability is based on the voluntary control of attentional focus, and it involves maintaining attention on the immediate experience, away from distractions such as self-referential thinking and mind wandering [79, 80]. This allows introspective awareness of mental processes and behavior, which is indispensable in the self-regulatory processes that support well-being [78].

Among these practices, mantra meditation from the Yoga tradition, Shamata and Vipassana meditation from the Buddhist tradition, self-inquiry from Vedanta, or secular variations like mindfulness or loving-kindness utilize several techniques and elements, like sounds or words, breath, body scan, external objects such as the flame of a candle, or simply observation of the present experience [75]. In general terms, all meditation techniques include the cultivation of focused, relaxed, and steady attention on the immediate experience in a state of nonjudgmental acceptance [42, 74, 75].

**Figure 1:**
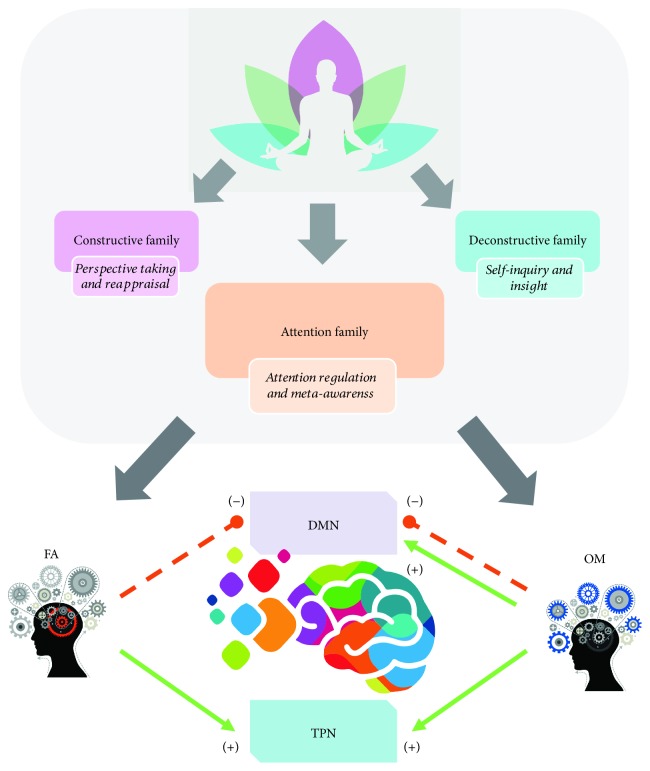
Typology of meditation practices and role of attentional family in the regulation of DMN and TPN networks. The traditional and contemporary meditation practices, following the classification of Dahl et al., can be grouped into attentional, constructive, and deconstructive families. According to this model, the primary cognitive mechanisms in these three families are (i) attention regulation and meta-awareness for the attentional family; (ii) perspective taking and reappraisal for the constructive family; (iii) and self-inquiry and insight for the deconstructive family. Attentional family practices teach a variety of processes related to the regulation of attention through 2 main techniques: focus attention (FA) and open monitoring meditation (OM) (see the text). Regardless of the used method, the activity of the default mode network (DMN) and task-positive networks (TPN) could be voluntarily modulated after the continuous training through their upregulation (green arrows) or downregulation (red-dotted arrows).

One of the most interesting classifications of the contemplative practices in neuroscience divides meditation in three super families, based on their primary cognitive mechanisms: (i) the constructive family, (ii) the deconstructive family, and (iii) the attentional family (Figure 1). Although they all use attention as a common ground, the first two are less related to this aspect of cognition. The first family is associated with repairing a maladaptive self-schema (perspective taking and reappraisal) and the second with exploration of the processes of perception, emotion, and cognition (self-inquiry) [75]. The third family is essentially concerned with the control of attention.

The attentional family potentiates the capacities to manipulate the orientation and aperture of attention and to disengage and reorient attention where it is needed. Hence, this family of practices could be summarized as focusing on training the ability to increase meta-awareness and diminish experiential fusion [75]. The attentional family is divided into two subcategories. The first is called focused attention (FA), and it involves attending to a chosen sensory or mental object (such as sounds, words, breath, images, thoughts, or even feelings), at the exclusion of everything else [41, 67]. In this practice, the participant remains attentive, and each time the mind begins to wander the person is trained to guide the focus of attention back with nonjudgmental awareness (Figure 1). The second type is called open monitoring (OM), which implies directing the attention to whatever arises in the field of consciousness; an unspecific focus is proposed for this technique, and the practitioner is instructed to be aware of everything that arises from the internal or external worlds [23, 41] (Figure 1).

Both FA and OM influence DMN activity [42]. Brewer et al. investigated the impact of FA meditation (concentration), OM meditation (choiceless awareness), and loving-kindness meditation (a member of the constructive family) on the DMN, showing that, in the three types of meditations, the main nodes of the DMN were deactivated in experienced meditators. The authors also found a strong coupling between the posterior cingulate, dorsal anterior cingulate, and dorsolateral prefrontal cortices, regions involved in self-monitoring and cognitive control during meditation [42]. In the case of FA meditation, the way attentional networks activate in response to the wandering mind in experienced meditators is known with some detail [67]. A follow-up resting state study has revealed that connectivity in attentional networks are directly associated with hours of practice, indicating that training in FA increases the attentional control of the practitioner in everyday life [67, 81].

OM is quite different from FA, because the practice is to observe thoughts and sensations while remaining unreactive, contributing to reduced emotional reactivity. The goal of OM is to understand the constant impermanence of reality while maintaining present awareness; some amount of mind wandering is allowed, but it is crucial to keep one's self disengaged from one's own train of thoughts. OM can be divided into two subtypes: one that focuses attention fully on the object (object-oriented OM) and those that focus attention on the quality of awareness during the experience (awareness-oriented OM) [75]. Investigations reporting the effects of OM meditation on the DMN have reported variable results, probably due to a lack of discrimination of these subtypes. While some publications indicate that OM, like FA, diminishes DMN activity [42, 81], it has also been reported that it results in an increased activation of the precuneus, a DMN hub, in contrast to FA [23, 82]. However, in both types of meditation a sustained effect in the control of the DMN occurs, beyond the period of practice, suggesting long-lasting effects in daily life [83]. Therefore, due to the fact that meditation, in general, is a mental training, it involves attention and the ability to maintain focus on a particular object. The effect of both types of meditation (OM and FA) could be interpreted as an increase in brain efficiency, promoted by their intensive training that improves the sustained attention and the cognitive control mechanisms as shown in several works [83, 84].

In experienced meditators of FA (as with OM), active control of task-positive brain regions are likewise observed, for example, in conflict monitoring, working memory, cognitive control, and emotional regulation, particularly through massive self-regulation in frontoparietal and insular areas. These regions and cognitive processes are relevant in cognitive decline, as well as in neurological and neuropsychiatric disorders [42, 82].

During selective attention, 7-14 Hz alpha rhythms are modulated in early sensory cortices, and this has been associated with an improved filtering of inputs to these brain areas [85, 86]. In attentional tasks, such as those guided by visual or somatic stimuli, a topographical distribution of alpha power reduction in the specific subregion associated with the task has been reported, as well as an increment in alpha power in unattended locations. This correlates with better performance in the task [87]. In meditation, particularly FA on body and breath-related sensations, enhanced modulation of 7-14 Hz alpha power is observed, which improves with increased hours of practice [88]. This modulation plays a key role in filtering inputs to the primary sensory neocortex and organizing the flow of sensory information in the brain [88, 89]. This top-down alpha rhythm modulation depends on the contextual cue, allowing the practitioner to better detect and regulate the wandering mind from its somatic focus, and could also be used to modulate pain sensation or compete with internal rumination in the case of depression [89].

Finally, it is not just the voluntary regulation of network connectivity that is associated with meditation, but also structural changes in the brain. A systematic review and meta-analysis [90] reports that certain brain structures are consistently altered when comparing meditation practitioners with control subjects. The majority of these regions are part of the DMN, as highlighted in a recent review [74]. Interestingly, there exists an anterior-posterior axis in the direction of the structural changes, with frontal and temporal areas reported to be thicker and parietal and occipital areas reported to be thinner in meditation practitioners versus controls [91]. Regions thicker in practitioners include areas involved in self-monitoring and integration of cognitive and emotional cues [92], reward processing, conflict monitoring, and self-regulation [93–95], memory (re)consolidation [88], and visual attention and perception [90, 91], in addition to exteroceptive and interoceptive body (meta)awareness [96]. Regions thinner in practitioners include areas involved in the processing of one's body in a spatial context [97], higher-order body image, self-related processing, and attentional shifting [91, 98]. However, it is not straightforward to interpret the direction of the alterations found. For instance, training that increases cognitive functions has been associated with decreased grey matter volumes [91, 99], putatively because of selective elimination of synapses [91, 100].

## 5. Integration of Basic and Clinical Research and Contemplative Practices in the Elderly

Despite the growing evidence, in basic and clinical research, focused on the study of the brain mechanisms underlying contemplative practices and their connection with clinically relevant outcomes, this body of research still faces major challenges with respect to the study, design, and practice classifications [101]. However, the effect of contemplative practices on network organization, specifically the DMN and TPN, has been related to neurological and neuropsychiatric diseases particularly relevant during senescence. If we consider that one-third of Alzheimer's disease is related to modifiable risk factors [102], such as certain negative psychoaffective states [103, 104], reducing this risk factor in 25% of individuals could prevent approximately three million cases of the disease worldwide [105]. Thus, it is reasonable to hypothesize that mental training for cognitive and affective regulation through contemplative practices has an important role in preventing cognitive decline, as well as neurocognitive disorders [74, 106, 107]. The effects of meditation on preserving age-related changes in cognitive functioning has been well established, specifically in areas such as the thalamus, insula, amygdala, hippocampus, and anterior cingulate cortex [108, 109].

Yoga and mindfulness meditation are two of the most studied contemplative practices, whose effects on aging we will detail below.

### 5.1. Yoga

Classic Yoga, or Raja Yoga, is a progressive method which includes different groups of practices aimed toward self-regulation [77]. This starts with ethics (when interacting with others), self-discipline, physical postures, and regulation of the breath, and ends in progressive states of sustained attention (withdrawal of the senses, concentration, meditation, and samadhi, a continuous state of meditative consciousness) [77]. This combination of practices allows, in a very low-cost experience, an improvement in lifestyle, constituting an integral preventive approach, as well as a complementary therapy to classical pharmacological approaches for mental diseases and diseases of aging. This is particularly pertinent when considering that polypharmacy is common in elderly people, and it increases the risk of drug interactions and adverse reactions up to 82% [110].

Yoga, like all contemplative practices, contains characteristics that promote the metacognitive capacity of the mind and self-regulation, including the potentiation of cognitive functions, such attention and memory in children and adults [111, 112], emotional regulation [113, 114], and prosocial behavior [113, 115]. A particular characteristic of the system is the modulation of both autonomic and cognitive aspects [77, 111–114, 116–120]. The practice of Yoga involves physical exercises (postures and regulation of breathing) to improve flexibility and strength, alongside cognitive training (meditation) to stimulate attention and meta-awareness [77]. Various sequences of postures derived from Hatha Yoga have been shown to have effects on stress and promote well-being [117]. The practice of postures involves constant short periods of focused attention on specific parts of the body, a kind of practice which could be classified differently to those previously described, called meditative movement [121]. Meditative movement includes forms of exercise that use movement, in conjunction with attention to body sensations, developing attention, proprioception, and a state of relaxation [77, 121]. The practice is followed by breathing exercises, such as alternate nostril breathing, which could aid in modulating autonomic responses and heart rate variability and improve memory and attention in adults [112, 119, 122–124]. The combination of these practices could improve the quality of life of its practitioners, fostering resilience and resistance and promoting healthy aging [117, 122, 125]. These exercises, together with ethical behavior and a positive attitude towards practice, including consistency and detachment from results (motivational/intentional factors), are the foundation and distinctive characteristic of the yogic practice [77].

A cross-sectional study in elderly female Yoga practitioners compared 21 women who had practiced Yoga for at least 8 years to 21 women naïve to Yoga, demonstrating that the first group showed a significantly greater brain cortical thickness in a left lobe cluster, including portions of the lateral middle frontal gyrus, anterior superior frontal gyrus, and dorsal superior frontal gyrus [126]. Furthermore, age-related decline in fluid intelligence was lower, while resting state functional brain networks were more resilient to damage. In addition, small-world brain architecture was stronger in long-term Yoga practitioners and meditation practitioners combined (16 of each practice type) compared to fifteen controls who were similar in age, education, exercise, and engagement in cognitive activities [127]. In an experimental study carried out by Vasudev et al., 24 elderly with depression were randomized to practice automatic self-transcending meditation based on mantra repetition (a form of FA) during a 12-week program, and were compared to 23 waitlist controls, with both groups receiving normal treatment for depression [128]. Depression and anxiety improved in meditation practitioners compared to treatment alone.

An eight-week meditation training, compared to relaxation training, resulted in decreased intraconnectivity in the DMN, salience network, and somatomotor network. Also, the meditator groups showed decreased connectivity strength between the DMN and other nodules (salience, frontoparietal, somatomotor, and visual networks) in tests for simple effects. There was lower nodal connectivity in the left posterior cingulate gyrus (associated with the DMN), bilateral paracentral lobule, and middle cingulate gyrus posttraining in the meditation group. No impact was observed on global level network organization in the resting state following this short-term intervention. In a separate study of the same participants, positive connectivity between the posterior cingulate cortex, precuneus, and the pons was observed in meditators [108, 129]. Lavretsky et al. randomly assigned elderly carers of family members with dementia to kirtan kriya yogic meditation (*n* = 23), another form of FA, or relaxation (*n* = 16), both interventions consisting in 12 minutes daily practice with audio recordings over 8 weeks, resulting in improved scores for cognitive functioning, Trails B, Mini-Mental State Examination, and mental health parameters (SF-36, Ham-D24) [130].

There are several meditation techniques that are part of yogic practice, mostly related to the attentional family, in particular FA [108, 129, 130], and may include body scan, attention on breath, and mantra repetition [131, 132]. However, despite the type of meditation, the practice of Yoga involves gradually sustaining more attention on the subject or perceiver of the experience than on the object of the experience (like mantra, breath, or body), and this meditation seeks to establish permanent self-awareness by separating the object of the experience from the subject or perceiver of the experience. This separation could include even the body, senses, and all kinds of thoughts and emotions of the practitioner, because all of these elements could be considered objects that can be detected by the perceiver or the subject [75, 133]. From this point of view, meditation together with Yoga techniques also has aspects of awareness-oriented OM [75, 133].

The DMN is involved in self-referential processes. Different types of meditation have the ability to activate a submodule of the DMN in Yoga practitioners [133]. Recently, it has been suggested that the DMN has three subnets, or operational modules: two symmetrical occipito-parieto-temporal subnets and one frontal subnet [134, 135]. An interesting aspect of this subdivision is that the frontal operational module is related to a sense of agency (the perceiver, the subject or witness of the experience) or a first-person perspective, and the other two modules are related to the continuity of the “I” embodied and localized within a bodily space [133–135]. Fingelkurts et al. compare the function of the DMN of healthy fully conscious subjects versus that of patients in vegetative and minimally conscious states, and they show that in vegetative patients all operative modules of the DMN are almost completely attenuated, while in patients with a minimal conscious state only the frontal subnet exhibits activity. Finally, in healthy and fully conscious subjects the three subnets function normally [134]. Interestingly, following long-term practice of three types of yogic meditation (such as focus on the breath, body scan, and mantra repetition), activity in the frontal operation module of the DMN is enhanced, while the other two modules are deactivated [133]. As in yogic meditation, it is always encouraged to avoid mind wandering and it is possible that the activation of the DMN following long-term practice is due to the constant self-referential process, and not due to mind wandering. This constant self-awareness could allow moving a safe distance away from the experience, which in turn could promote self-regulated behaviors, improve mental health [75], and foster a healthy lifestyle [108, 129].

Moreover, meditation is able to modulate another network that could be fundamental to self-referential processes: the paralimbic network that involves the anterior cingulate and medial prefrontal cortices [136, 137]. In experienced practitioners, Yoga nidra meditation, a type of body scan meditation, is accompanied by a strong increase in sensory awareness [136] and significant differences in the brain network activity as compared to the resting state [136, 137]. In contrast, sustained activity in the paralimbic network constituted by the midline frontal regions is observed in both meditative and resting states. This finding has led to the hypothesis that the paralimbic network contributes to the regulation of a common reference of self-perspective [136, 137]. Taken together, these data could indicate that attention, awareness, and emotion are integrated by a paralimbic network, and the activity of this region, together with the DMN, could be modulated by meditation techniques derived from Yoga. This kind of regulation could be helpful to efficiently allocate brain resources to optimize behavior and well-being.

Also, the characterization of each of the subnetworks of the DMN, and its relation with other networks (including the paralimbic network) in various contemplative practices, such as Yoga in experimented practitioners, could be a novel methodological approach to better understand the role of the DMN in meditation. At the same time, the study of the DMN and meditation may provide greater insights into the operational module involved in the regulation of the DMN, and its relationship with several neuropathologies related (or not) with aging.

### 5.2. Mindfulness

Mindfulness has its origin in the Buddhist tradition [2, 138], and it may be understood as a mental state characterized by “paying attention on purpose, in the present moment, and nonjudgmentally to the unfolding of experience, moment by moment” [86]. It may be considered a key skill developed through contemplative practices such as meditation and Yoga [77, 139]. The benefit delivered by this practice lies on the notion that attention is a core function to be developed [140, 141]. Its underlying neural mechanisms would be related to the salience network [141, 142], the executive control network, and the orienting network [141, 143].

Several authors have proposed that mindfulness training may help older adults in managing the cognitive, emotional, and psychological challenges of aging [144–146]. This has been corroborated by a meta-analysis of 47 trials that demonstrated a positive impact of mindfulness meditation programs over anxiety- and depression-related outcomes, independent of age [147]. Indeed, Prakash et al. showed that mindfulness disposition, defined as the ability to focus on the present and disengage from DMN-related thinking, is associated to enhanced connectivity in the DMN in older adults [148]. Gard et al. assessed mindfulness using the Five Facet Mindfulness Questionnaire, across all participants, in a cross-sectional study including meditation (insight meditation from the deconstructive family) and Yoga practitioners (Kripalu Yoga, a traditional Raja Yoga school). They found that this dimension related positively to fluid intelligence, network resilience, and integration [127]. This suggests a mechanistic role of meditation in the preservation of intelligence and functional brain network architecture [127]. In addition, a randomized controlled trial, conducted by Wells et al., corroborated that a mindfulness-based stress reduction program, which applied OM techniques (weekly 2-hour group sessions over 8 weeks, with suggested 30-minute daily home practice), resulted in an improved connectivity in the posterior cingulate cortex, medial prefrontal cortex, and left hippocampus in participants who suffered from mild cognitive impairment, as compared to controls who did not receive this intervention [149]. This practice demonstrated an enhancement in executive function and greater leftward frontal alpha asymmetry in elderly practitioners [150]. Other findings supporting mindfulness meditation have verified that a daily practice, escalating from 5 to 20 minutes over 6 weeks, is linked to grey matter changes (i.e., increases in the right precuneus and decreases in the left prefrontal cortex, right hippocampus, right thalamus, and right parietal cortex [151]). Similarly, Laneri et al. analysed the effects of long-term mindfulness meditation on the brain's white matter microstructure and its aging by employing diffusion tensor imaging in 33 meditators with 5-38 years of experience from Buddhist and Zen centres in Germany (all meditators practiced mindfulness meditation styles such as Vipassana, of the deconstructive family, Shamatha, and Zazen), and in 31 healthy nonmeditators [109]. The authors concluded that mindfulness meditation might contribute to the preservation of the integrity of white matter, diminishing age-related white matter degeneration.

Following the model of attention that proposes three main components (alerting, orienting, and conflict resolution, or an executive component) [74], elderly randomized to mindfulness training (10 minutes, 5 times a week for 8 weeks) exhibited improved response latency in the Stroop test and in electrophysiological measures, indicating improved maintenance of goal-directed attention in this visuospatial task [141]. However, other authors found no improvement in measures of executive control and emotional regulation [12]. On the other hand, a decline of efficiency, specifically in the executive component of attention, was observed in elderly meditators compared to meditation-naïve, young adults [80]. This effect was not observed in age-matched long-term meditators, with similar education level and cognitive function (*n* = 16 in each group) [80], where 12 participants practiced the Zen tradition and four practiced the Tibetan tradition (both traditions emphasize OM techniques). This highlights the protective role of long-term meditation on the decline of the executive component of attention in aging. Mindfulness meditation, based on mindfulness-based cognitive therapy and mindfulness-based stress reduction (both corresponding to the OM type of attentional family), applied with a weekly personalized training and home practice for over two months, did not result in changes in cognitive or physiological outcome measures in 66 elderly with stress randomized to this intervention, compared to 68 waitlist controls. However, self-rated measures related to negative affect and stress showed improvement, and a significant change in the neuroticism dimension was observed [152]. In addition, no differences in mood or cognitive outcomes were found in 8 elderly participants who completed 1 hour of weekly internet-based mindfulness meditation sessions (a standardized and structured program that uses mindfulness-based cognitive therapy and mindfulness-based stress reduction), with 30 minutes of daily practice for 6 weeks, as compared to 8 controls who completed a wellness and education program in a similar format, over the same period [153]. However, this was a pilot study, with online delivery of the intervention, and findings should be appraised with caution due to the low number of participants included and potential selection and performance biases.

These data collectively suggest that the use of meditation could increase the optimal function of the brain in the elderly through voluntary regulation of brain networks, such as the DMN and TPN. This is evidenced not only by functional changes, but also structural ones, which could facilitate optimal aging and prevent neurological and neuropsychiatric diseases. A greater part of these studies, developed using standardized techniques, highlight the impact of mindfulness OM techniques as a promising practice to enhance cognitive functioning during aging.

## 6. Perspectives

**Figure 2:**
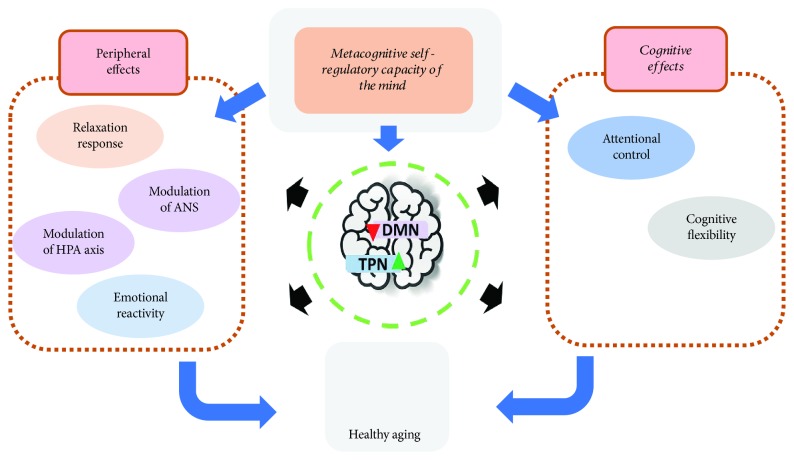
Model of meditation effects to promote optimal aging. The constant practice of attentional family meditation develops the metacognitive self-regulatory capacity of the mind. This consists in the voluntary control of attentional focus and keeping the attention in the present experience, without self-referential thinking and mind wandering. This metacognitive ability can modulate cognitive, emotional, behavioral, and autonomic output. The question is: Can these skills promote a healthy aging? ANS: autonomic nervous system, HPA: hypothalamic-pituitary-adrenal axis.

While more clinical and basic research is needed to establish the modulation of the DMN and TPN through meditation, and to understand the impact of modulation on aging and mental disease, the data indicate that meditation may influence different cognitive processes, thus increasing attentional focus and cognitive flexibility [154]. Meditation is also suggested to modulate several other physiological processes, including enhancing relaxation through the decrease of sympathetic nervous system activity, controlling the hypothalamic-pituitary-adrenal axis in stress, decreasing heart rate variability, and diminishing emotional reactivity [145, 155–157]. All these processes are mediated through what Dorjee calls “the metacognitive self-regulatory” capacity of the mind, an ability based on meta-awareness and sustained attention [78] (Figure 2). Furthermore, these findings suggest that it is plausible to develop new strategies to facilitate optimal aging.

Nevertheless, the mechanisms by which meditation modulates the DMN and TPN, and how this influences physiological processes, remain elusive. At the neural level, meditation has been shown to both increase and decrease specific brain networks. In general, meditation decreases connectivity between the DMN and salience, frontoparietal, somatomotor, and visual networks, while increasing connectivity in the posterior cingulate cortex, medial prefrontal cortex, and left hippocampus. It is important not to think that these differences of direction in connectivity modulation imply an inconsistent result: given that both networks are involved in different processes, this opposed connectivity could shed light on the mechanisms involved in the effects of meditation on cognitive processes. Further research is needed in order to elucidate what is the role of these specific networks in the specific cognitive processes shown to be modulated by meditation.

But, indeed, this active cognitive process which is able to modulate several brain networks has been suggested as a therapeutic approach for neuropsychiatric and even neurodegenerative disorders, important for an optimal aging. Interestingly, and as suggested by this article, many of these symptoms could be reduced directly through the modulation of DMN activity that could interfere with goal-directed tasks [158, 159]. We also have shown that within the current classification of meditation, the attentional family has a greater capacity for the modulation of the DMN. Also, the greater the hours and frequency of practice (experience), the greater is the decrease in the activity of the DMN during the task [160]. This agrees with the general idea that during goal-directed tasks, there could be an anticorrelation between the attentional networks and the DMN. In this context, FA meditation has shown several positive results, while OM meditation has divergent results regarding control of the DMN in the literature.

At present, the divergence of the results about this type of meditation is an open question. However, it is necessary to consider an important factor about the nature of the OM meditation. Unlike FA meditation, which is directly associated with an object of attention (either an internal or external goal-directed task), to achieve a state of effortless awareness, OM meditation increases the aperture of awareness to all mental objects and sensory inputs that spontaneously appear and disappear from the mental field, to allow a meta-awareness of one's own mental habits [160]. This practice therefore requires a greater skill on the part of the practitioners, due to its similarity with a state of mental wandering. As we previously described in this article, OM meditation demonstrates similarities and possibly an interaction with certain nodes of the DMN, and possibly with the wandering of the mind [160].

Thus, it is possible that the activation of the DMN during the practice could be related to the constant self-referential process, and not due to mind wandering, as we previously mention in the Yoga section. To see in detail the difference between the state of OM and mind wandering, refer to Vago and Zeidan [160].

Also, another possible explanation to the divergence in the results of this type of meditation may be due to some extent in the complexity of the proposed task and the inability of practitioners, especially novices, to achieve this skill. This is especially relevant when OM meditation is instructed as a primary technique, without the achievement of previous stages (such as focused attention on an object), going in an opposite direction to the progressive path in which the eastern schools of meditation have taught for centuries [77, 160].

In this context, Yoga as a meditation school seems to have a more integral approach because of the particular dedication that Yoga puts into the gradual progression of meditative practice. One of the distinguishing characteristics of classical—or Raja—Yoga is the combination of practices like postures, breath control, and several forms of meditation that could modulate the autonomous nervous system, principally by postures and breathing exercises. This modulation is a door to the regulation of other important systems such as immune, digestive, cardiovascular, or muscular systems. Unlike other practices that integrate movement, attention, and breathing, and that could promote similar effects (for example, Tai-Chi or Qi gong, which have also been the focus of scientific interest [121, 161]), Yoga could also modulate cognitive functions like attention, memory, and mind wandering, due to the inclusion of specific techniques of sustained attention/meditation. In this context, Yoga meditation also includes techniques that could be classified as FA and OM meditation, which in turn promote the activation of TPNs and also modulation of the DMN [101, 133]. However, more research and a broader classification is required to include all the meditation techniques present in Yoga, because the current classification of meditations is eminently directed for those belonging to the school of mindfulness.

In this context, another possibility could be to organize these types of disciplines under a general concept that may include most of the practices that are in the current investigation. These practices must accomplish basic characteristics, such as those proposed by Dorjee to be the metacognitive self-regulatory capacity of the mind, based on meta-awareness and sustained attention [78] (Figure 2). This allows (i) integrating different methods, (ii) providing a better characterization of the techniques, interventions, and results, and (iii) avoiding control over concepts by one or two schools at the exclusion of others. While several techniques or schools that could be characterized as contemplatives have different approaches (two of the best known are Yoga and mindfulness), there are many others, including the relaxation response, the Liverpool model of mindfulness, or other meditative moment techniques such as Tai Chi [72, 121].

So, it is fundamental to increase and systematize the research in contemplative practices, covering the different aspects of each practice that could causally explain its cognitive and neurophysiological effects, and how this practice could promote well-being at different stages of life (crucially, in aging), as well as how it could be used in the prevention and treatment of several types of diseases.
